# Transcriptomic dataset of wild type and *phoP* mutant *Pectobacterium versatile*

**DOI:** 10.1016/j.dib.2020.106123

**Published:** 2020-08-02

**Authors:** Natalia Gogoleva, Uljana Kravchenko, Yevgeny Nikolaichik, Yuri Gogolev

**Affiliations:** a Kazan Institute of Biochemistry and Biophysics, Federal Research Center "Kazan Scientific Center of RAS", Kazan, Russia; b Department of Molecular Biology, Belarusian State University, Minsk, Belarus

**Keywords:** *Pectobacterium versatile*, RNA sequencing, Transcriptome, PhoP, PhoQ

## Abstract

RNA-Seq transcriptome data for the wild type and *phoP* mutant strains of *Pectobacterium versatile* is described. *P. versatile* is a recently introduced name for a species of plant pathogenic bacteria that unites a group of strains previously embedded within the *Pectobacterium carotovorum* clade [1,2]. Little detail is available about how this pathogen adapts to changing environmental conditions, including those within its host plant. The PhoP/PhoQ two-component system is an important sensor responding to several stimuli and is present in most species of enteric bacteria. It usually controls large regulons, which vary greatly even between closely related species [3]. This dataset enables the discovery of the genes under direct or indirect transcriptional control by PhoP in *P. versatile* and should help to understand the physiology of this plant pathogen.

**Specifications Table**

**Table:** 

Subject	Biochemistry, Genetics and Molecular Biology (General)
Specific subject area	Molecular Biology
Type of data	Transcriptome sequences
How data were acquired	Illumina HiSeq 2500 sequencing platform
Data format	Raw Illumina data in FastQ format
Parameters for data collection	Wild type and *phoP* mutant *Pectobacterium versatile* cultures grown in synthetic media to mid-log phase
Description of data collection	mRNA was extracted from eight independent cultures (four wild type and four mutant) and subjected to cDNA sequencing
Data source location	Belarusian State University, Minsk, Belarus
Data accessibility	Repository name: NCBI Sequence Read Archive Data identification number: PRJNA627079 Direct URL to data: https://www.ncbi.nlm.nih.gov/bioproject/PRJNA627079

Value of the Data

• This dataset is, to our knowledge, the first RNA-seq one for *P. versatile* and will be valuable for the *Pectobacterium* sp. research community for characterizing the highly divergent regulon controlled by the global transcription factor PhoP.
• The data may be useful for researchers studying the adaptation of *P. versatile* to changing environment, including plant colonisation.
• The data can be used to define PhoP regulon and to establish PhoP role in the control of *P. versatile* virulence.
• This dataset can be used to study operon organisation in *P. versatile.*

## 1 Data description

**Fig. 1:**
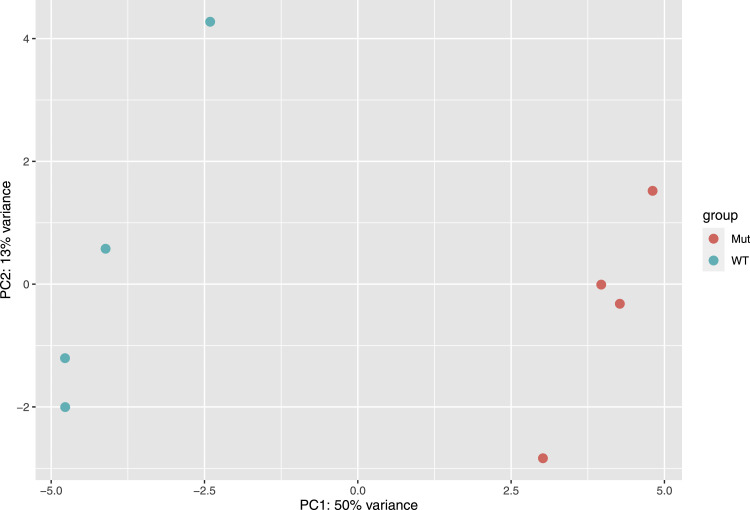
Principal component analysis (PCA) of the general transcriptome characteristics. The first principal component (PC1) accounted for 50% and the second principal component (PC2) for 13% of the total variance in the dataset. Legend description: “WT”– samples of cultures of *P. versatile* strain JN42, wild type; “Mut” – samples of cultures of the UK1 strain, *phoP* insertional mutant of JN42.

The dataset contains sequencing data obtained through the transcriptome sequencing of two *P. versatile* strains: JN42 and its *phoP* mutant derivative UK1 grown in the synthetic medium supplemented with polygalacturonic acid. Samples for transcriptome profiling were collected at the exponential growth phase. FASTQ files were deposited in NCBI Sequence Read Archive and are accessible through the BioProject PRJNA627079. Information about bacterial culture samples, statistics of sequence reads and sequence coverage data is shown in Table 1. PCA plot of RNA-seq data presented in Fig. 1 demonstrates the variance between sample groups and sample replicates according to gene expression levels. Each dot in the Fig. 1 indicates a particular sample.

**Table 1:** Details of RNA-seq data submitted to the NCBI Sequence Read Archive (SRA).

				Reads	
Strain	Sample ID	Biosample accession no.	SRA accession no.	Total number	Mapped to reference
JN42 (wild type)	wt_rep1	SAMN14651075	SRR11581681	11006472	99.16%
	wt_rep2	SAMN14651076	SRR11581680	9867857	99.08%
	wt_rep3	SAMN14651077	SRR11581679	11415728	99.18%
	wt_rep4	SAMN14651078	SRR11581678	11835173	99.15%
UK1 (*phoP* mutant)	phoP_rep1	SAMN14651079	SRR11581677	7841926	99.12%
	phoP_rep2	SAMN14651080	SRR11581676	10640935	99.11%
	phoP_rep3	SAMN14651081	SRR11581675	10833222	99.12%
	phoP_rep4	SAMN14651082	SRR11581674	10780714	99.07%

## 2 Experimental design, materials and methods

### 2.1 Bacterial strains and growth conditions

*P. versatile* strains JN42 (wild type) and UK1 (*phoP* insertional mutant of JN42) were used and grown in minimal medium composed of K_2_HPO_4_ (10,5 g/l), KH_2_PO_4_ (4,5 g/l), (NH_4_)_2_SO_4_ (1 g/l), sodium citrate (0,6 g/l), 0.5 mM MgSO_4_, 10 µM CaCl_2_, 0.2% glycerol and 0.5% Sodium polypectate (Sigma). For RNA isolation, four separate cultures of each strain were grown at 28 °C with aeration (180 rpm) to mid-log phase (OD600 = 0.4).

### 2.2 RNA isolation, cDNA library preparation and sequencing

Bacterial cells in mid-log phase cultures were fixed by adding phenol/ethanol (1/20 v/v) solution to 20% and kept on ice for 30 min. The fixed cells were harvested (8000 g, 5 min, 4 °C) and resuspended in 1 mL of ExtractRNA Reagent (Evrogen, Russia) and the subsequent procedures were performed according to the manufacturer's instructions. Residual DNA was eliminated by treatment of RNA samples with DNAse I (Thermo Fischer, USA). Total RNA was processed using Ribo-Zero rRNA Removal Kit (Gram-Negative Bacteria) (Illumina, USA) and NEBNext Ultra Directional RNA Library Prep Kit for Illumina (NEB, USA) according to manufacturer's instructions. The quality and quantity of the cDNA libraries during processing before sequencing were monitored using the Agilent 2100 Bioanalyser (Agilent Technologies, USA) and CFX96 Touch Real-Time PCR Detection System (Bio-Rad Laboratories, USA). Sequencing was conducted by a HiSeq 2500 Sequencing System (Illumina) at Joint KFU-Riken Laboratory, Kazan Federal University (Kazan, Russia).

### 2.3 Sequence QC and filtering

84,222,027 reads were obtained in total with a length of 57 nucleotides (Table 1). FastQC software (Version 0.11.5) [4] was used to assess the quality of the raw Fastq files and clean reads. Raw reads were filtered using BBDuk (v. 37.23, http://jgi.doe.gov/data-and-tools/bb-tools/) to remove Illumina adapters, NEB indexes and to quality-trim right end to Q20 (ktrim 1⁄4 r k 1⁄4 23 mink 1⁄4 11 hdist 1⁄4 1 tpe tbo minlen 1⁄4 25 qtrim 1⁄4 r trimq 1⁄4 20). Thereafter, the rRNA reads were eliminated by using SortMeRNA v2.1 program [5]. DESeq2 [6] was used to assess variance between sample groups and sample replicates using principal component analysis (PCA). PCA plot shown in the Fig. 1 demonstrates the overall quality of our sample collection, library preparation, and sequencing.

### 2.4 Reads alignment to the reference genome

The reads were mapped onto the genome sequence of *P. versatile* strain 3-2 (GenBank accession CP024842) which is the wild type parent of the laboratory strain JN42. BWA version 0.7.16a [7] was used to build the index of the reference genome and align the reads to the reference genome with default aligner parameters. SAM files of alignments created by BWA were converted to sorted BAM files with SAMtools v. 1.10 [8] using samtools sort command. Reads mapping statistics are presented in Table 1.

## Author's contribution and ethics statement

Natalia Gogoleva: Investigation, Methodology. Uljana Kravchenko: Investigation, Software. Yevgeny Nikolaichik: Conceptualization, Data curation, Writing - Original draft preparation, Review & Editing. Yuri Gogolev: Conceptualization, Supervision, Writing - Original draft preparation, Review & Editing, Funding acquisition. All ethical requirements for such studies were observed in the preparation of the publication. The work was not related to the use of human objects and did not include experiments with animals.

## Declaration of Competing Interest

The authors declare that they have no known competing financial interests or personal relationships which have, or could be perceived to have, influenced the work reported in this article.

## Appendix Supplementary materials

Image, application 1
